# Bilothorax: A Bitter Complication of Liver Surgery

**DOI:** 10.1155/2013/372827

**Published:** 2013-03-03

**Authors:** L. A. C. Hamers, K. Bosscha, M. H. van Leuken, M. A. M. Moviat, C. P. C. de Jager

**Affiliations:** ^1^Department of Intensive Care Medicine, Jeroen Bosch Hospital, Henri Dunantstraat 1, 5223 GZ Den Bosch, The Netherlands; ^2^Department of Surgery, Jeroen Bosch Hospital, Henri Dunantstraat 1, 5223 GZ Den Bosch, The Netherlands; ^3^Department of Radiology, Jeroen Bosch Hospital, Henri Dunantstraat 1, 5223 GZ Den Bosch, The Netherlands

## Abstract

Bilothorax is a rare condition, mostly associated with surgery involving the biliary system or trauma. In this article a case of bilothorax secondary to liver surgery is reported, which recovered by pleural and abdominal drainage. Bilothorax should be considered as a cause of respiratory detoriation in patients with recent biliary or hepatic surgery.

## 1. Introduction

Complications of liver surgery are mainly related to infection or bile leakage. Bilothorax is a rare condition, mostly associated with surgery involving the biliary system or trauma. We report a case of bilothorax secondary to liver surgery, which was conservatively treated by pleural and abdominal drainage, with uneventful recovery.

## 2. Case Report

A 58-year-old woman with a medical history of a colonic carcinoma was surgically treated two years ago by means of a left hemicolectomy, together with resection of the spleen and the adrenal gland. The postoperative period was complicated by ARDS. Adjuvant chemotherapy was started because of liver metastasis, followed by metastasectomy of liver segment 8, eight months after primary surgery.

**Figure 1:**
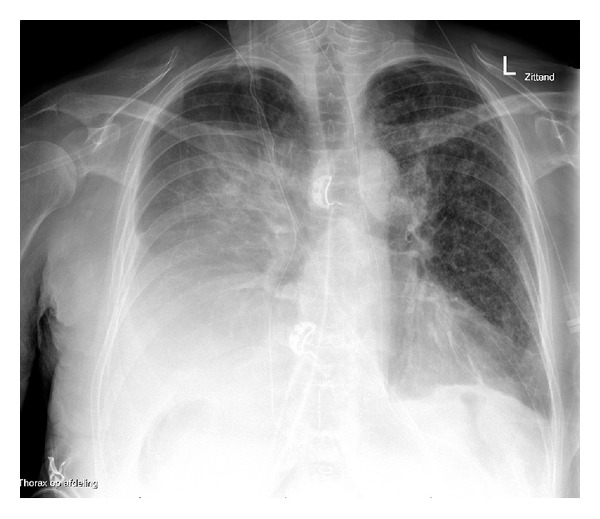
Chest radiography showing unilateral pleural effusion.

Two years later, because of progression of liver metastases on regular follow up CT scans, metastatsectomy was carried out again, this time affecting liver segments 5, 6, and 8. Additionally, segment 4 was treated by radiofrequency ablation. Although preoperatively a diaphragm leak was suspected, this could not be proven. Four days postoperatively, she developed respiratory insufficiency (SpO2 84% without oxygen, 94% supported by 14 litres nonrebreathing mask, respiratory rate 20/minute). A chest X-ray showed a pleural effusion halfway up the right hemithorax (see Figure 1). She was admitted to the intensive care unit. 

A right thoracostomy tube was inserted which immediately drained large amounts of yellowish fluid (2 litres in total). The suspicion of a bilothorax was confirmed by a high concentration of bilirubin (355 *μ*mol/L). The same day, an abdominal drain was inserted ultrasound guided in the right upper quadrant, which produced bile as well (approximately 200 cc/day). Subsequently, bile production of the thoracostomy tube ceased, and it was removed on postoperative day 7. Because cultures of the drained fluid remained negative, empiric antibiotic therapy was stopped after 5 days. ICU course was complicated by ARDS on postoperative day 5, for which mechanical ventilation was required, and corticosteroids were started. On the 13th postoperative day, patient was extubated and could be discharged to the regular ward.

Patient was readmitted to the intensive care unit on postoperative day 19 with haemodynamic and respiratory instability due to abdominal sepsis. The pleural cavity showed no new massive effusion except from minimal bilateral reactive fluid. An abdominal CT can showed a large effusion in the subcapsular liver compartment at the side of the metastasectomy, interpreted as a septic hemorrhage. A bigger tube was inserted and drained old blood. *Enterococcus faecalis* was cultured from the abdominal fluid and blood, for which amoxicillin was started 7 days after moment of culture. As soon as clinical condition permitted, an ERCP was performed with biliary decompression using a 7 French synthetic stent. On day 49 she left the hospital in a fairly good condition, still with an abdominal drain in situ just producing some yellowish fluid. 

## 3. Discussion

Bilothorax is rarely seen nor described in the literature, mostly reported after traumatic or surgical injury of the liver and/or biliary system.

Several mechanisms for bile entering the pleural cavity have been put forward. From observations it can be concluded that air or fluid can traverse the diaphragm by direct passage through a congenital, traumatic, or erosive defect. A supposed indirect route is following the connective tissue sheaths of the oesophagus and great vessels into the mediastinum, with breakthrough to the pleural cavity [1]. In this case, however, obviously a diaphragm defect had been caused during surgery. 

Moreover, as any fluid, bile will go the path of least resistance. Therefore, a subsequent bilothoracic fistula only develops when intra-abdominal accumulation of bile is inadequately drained. Consequently, when the biliary system was drained by an intra-abdominal tube in our patient, bile leakage into the pleural cavity ceased and the chest tube could be removed without recurrence of biltohorax.

The respiratory status of a patient can be rapidly compromised by a bilothorax. Not only lung volume is decreased by the fluid, also, bile causes an inflammatory response due to a directly corrosive effect on the pleural layers. In our patient, this may have resulted in ARDS. Bile acids have been implicated in aspiration related ARDS [2]. However, only one case of bilothorax as precipating factor has been reported to our knowledge [3]. Our patient obviously had already increased sensitivity for developing lung injury, by means of active malignancy, ongoing biliary leak shortly after surgery, and the previous episode of ARDS as a primary insult.

 Bilothorax can be diagnosed relatively easily. When chest X-ray reveals a pleural effusion shortly after presumptive liver or biliary damage, an agressive approach should be chosen and an early punction has to be performed. The fluid will easily be recognized as distinctive, and chemical analysis confirms the diagnosis of bilothorax. No extensive experience in treatment of bilothorax exists. Several reports describe conversative management by means of a tube thoracostomy as a succesfull approach [4, 5]. Our case illustrates the importance of adequate drainage of bile via abdominal route, since biliopleural leakage may be maintained otherwise.

In conclusion, bilothorax should be considered as a cause of respiratory detoriation after biliary or hepatic surgery or trauma. Immediate drainage via chest thoracostomy is obligatory. Additionally, we emphazise the importance of adequate bile drainage also via abdominal route. Reasonably, bilothorax was the precipitating agent for ARDS in our patient.
